# Remote APOE counseling and genotyping via telehealth: Early findings from a new care delivery model in the United States

**DOI:** 10.1002/alz.095786

**Published:** 2025-01-09

**Authors:** Anitha Rao, Leyla Anderson, Travis Wilkes, Steven R Verdooner

**Affiliations:** ^1^ NeuroVision Imaging, Inc, Sacramento, CA USA

## Abstract

**Introduction:**

APOE (apolipoprotein E) genotyping determines an individual’s risk of developing Alzheimer’s disease and unique pathological characteristics vital to treatment consideration. The presence of the ε4 allele is considered a dose‐dependent risk factor for late‐onset Alzheimer’s disease, with each additional copy of the allele adding to the risk. Genetic counseling and education are essential as disclosure can lead to psychosocial issues, employment issues, and family stress. Traditionally, genetic counseling for Alzheimer’s disease has occurred during face‐to‐face encounters, thereby marginalizing patients who live in rural and underserved areas with high social determinants of health. In this study, we evaluated the application of telehealth services for APOE‐specific genetic counseling with an emphasis on solutions‐based education promoting health literacy and equity.

**Methods:**

Participants were enrolled into the program from an electronic referral process from a primary care or neurology outpatient practice. A HIPAA‐secure and encrypted electronic health system was used to conduct the telehealth visits. Consent was collected from all patients. A BrainHealth Educator (BHE), typically an RN, was trained on best practices and protocols on APOE testing from resources such as AGREEDementia.org. BHEs conducted pre‐ and post‐test education and counseling. In addition, BHEs coordinated logistics of at‐home buccal swab self‐collection and sample transportation to lab.

**Results:**

A total of 29 patients enrolled in the genotyping program with 22 females and 7 males. Each patient had a 30‐minute pre‐test education that reviewed Mendelian inhertiance, APOE polymorphism combinations, and evidence based risk stratification for developing Alzheimer’s disease. All possible genotypes were represented amongst the study subjects (n = 29): E2/E2 (1), E2/E3 (3), E2/E4 (2), E3/E3 (12), E3/E4 (7), E4/E4 (1), pending (3).

**Conclusions:**

Advancements in cognitive care are rapidly growing and there is a need for genetic risk assessment and stratification. Ordering genetic testing requires adequate pre‐test counseling to ensure appropriate timing and utilization. The interpretation and dissemination of genetic information requires structured support from a trained healthcare professional skilled with providing solutions‐based guidance. Under these circumstances, individuals can receive APOE‐specific genetic health information in a manner that enhances cognitive care planning, prevention, and treatment with targeted monoclonal antibody treatments.

